# The Clinical Impact of Low-Volume Lymph Nodal Metastases in Early-Stage Cervical Cancer: The Senticol 1 and Senticol 2 Trials

**DOI:** 10.3390/cancers12051061

**Published:** 2020-04-25

**Authors:** Benedetta Guani, Vincent Balaya, Laurent Magaud, Fabrice Lecuru, Patrice Mathevet

**Affiliations:** 1 Gynecologic Department, CHU Vaudois, 1011 Lausanne, Switzerland; 2 Faculty of Biology and Medicine, University of Lausanne, 1015 Lausanne, Switzerland; 3 Faculty of Medicine, University of Paris, 75006 Paris, France; 4 Epidemiology and Research Department, Hospital of Lyon, 69003 Lyon, France; 5 Faculty of Medicine, University of Lyon, Claude Bernard Lyon 1, F-69008 Lyon, France; 6 Faculty of Medicine, University of Saint-Étienne, F-42023 Saint-Etienne, France; 7 Breast, Gynecology and Reconstructive Surgery Department, Curie Institute, 75248 Paris, France

**Keywords:** cervical cancer, micrometastases, sentinel lymph node

## Abstract

*Background:* With the development of the sentinel node technique in early-stage cervical cancer, it is imperative to define the clinical significance of micrometastases (MICs) and isolated tumor cells (ITCs). Methods: We included all patients who participated in the Senticol 1 and Senticol 2 studies. We analyzed the factors associated with the presence of low-volume metastasis, the oncological outcomes of patients with MIC and ITC and the correlation of recurrences and risk factors. *Results:* Twenty-four patients (7.5%) had low-volume metastasis. The risk factors associated with the presence of low-volume metastasis were a higher stage (*p* = 0.02) and major stromal invasion (*p* = 0.01) in the univariate analysis. The maximum specificity and sensitivity were found at a cutoff of 8 mm of stromal invasion. In multivariate analysis, the higher stage (*p* = 0.02) and the positive lymphovascular space invasion (*p* = 0.02) were significantly associated with the MIC and ITC. Patients with low-volume metastasis had similar disease-free survival (DFS) (92.7%) to node-negative patients (93.6%). The addition of adjuvant treatment in presence of low-volume metastasis did not modify the DFS. *Conclusions:* These results confirm our previous analysis of Senticol 1: the presence of low-volume metastasis did not decrease the DFS in early-stage cervical cancer patients.

## 1. Introduction

In early-stage cervical cancer, the status of the lymph nodes is one of the most important prognostic factors. The revised International Federation of Gynecology and Obstetrics (FIGO) 2018 classification [1] specifically defined lymph node involvement as a stage IIIC disease and highlighted the importance of lymph node metastasis as a major prognostic factor in cervical cancer. The systematic ultrastaging of sentinel lymph nodes (SLNs) has frequently led to diagnoses of isolated tumor cells (ITCs) or micrometastases (MICs). Macrometastases (MACs) are the lymph nodal metastases > 2 mm, MICs are the lymph nodal metastases > 0.2 mm and up to 2 mm, ITCs are tumor cells clusters < 0.2 mm at the largest diameter in the nodal zone. In contrast to MACs, the presence of ITCs does not imply a change of stage, according to the new FIGO 2018 classification [1]. At present, the impact of low-volume metastasis, defined as the presence of MICs and ITCs, remains unclear. 

In literature, numerous retrospective studies have assessed the impact of MIC and ITC on oncologic outcomes [2,3,4], but only two prospective studies can be found [5,6]. In the retrospective studies [2,3,4], the presence of MIC was significantly associated with decreased overall survival. In the study by Cibula [2], MIC had a similar prognosis to that of MAC, but ITC had no prognostic significance. Only one retrospective study [7] did not demonstrate an association between the presence of MIC and recurrence or overall survival.

On the contrary, our prospective analysis of Senticol 1 published in 2019 [5] demonstrated that neither MICs nor ITCs had an impact on the risk of recurrence or on survival after a follow-up of three years. Our results were confirmed by a recent 2019 prospective study by Nica [6].

The uncertain significance of low-volume metastasis has, so far, justified the use of adjuvant therapy and ultrastaging. According to the ESGO/ESTRO/ESP Guidelines 2018 [8], the metastatic involvement of the pelvic lymph nodes, including the presence of MAC or MIC in either sentinel nodes or any other pelvic lymph nodes as detected by intraoperative or final pathologic assessment, justifies adjuvant treatment for chemoradiotherapy. There are no clear guidelines for ITC.

Unfortunately, as stated in a recent review [9], the data are too scarce to conclude whether the diagnosis of MIC or ITC should influence treatment decisions, as is the case for MAC. 

In order to add data to the currently limited literature, we analyzed the impact of low-volume metastasis through two French prospective multicenter studies, Senticol 1 [10] and Senticol 2 [11]. 

## 2. Results

Overall, 321 patients fulfilled the inclusion criteria and were included in this study, ultrastaging analysis was performed for all non-sentinel lymph nodes (NSLN) in 141 patients (44% of patients). For the remaining patients, ultrastaging was only performed for SLN.

### 2.1. Lymph Node Metastases

Forty-one patients out of whole study group (12.7%) had positive lymph node tests (MAC, MIC, and ITC). Twenty-four patients (7.5%) had low-volume metastases: in 22 cases, low-volume metastases were found in SLN and in two other cases in NSLN. They were divided into MIC in 11 cases and ITC in 13 cases. Among NSLN, one ITC case in a false negative SLN and one in a patient with SLN detection failure were found. We also found 17 MAC. All other patients (280) had no nodal metastases (Table 1). 

Following the 2007 letter to the editor from Ferenczy [12] warning about the risk of false positives SLNs due to the confusion of non-tumor cells with ITCs, we analyzed the association of ITCs (detected by himmunohistochemistry in addition of HE examination) with tumor size, the presence of a diagnostic conization, and preoperative brachytherapy. No statistically significant associations were found. We therefore estimated that the ITCs found in our study are genuine isolated tumor cells.

### 2.2. Factors Associated with Low-Volume Metastases

We analyzed the factors associated with the presence of low-volume metastasis.

**Figure 1:**
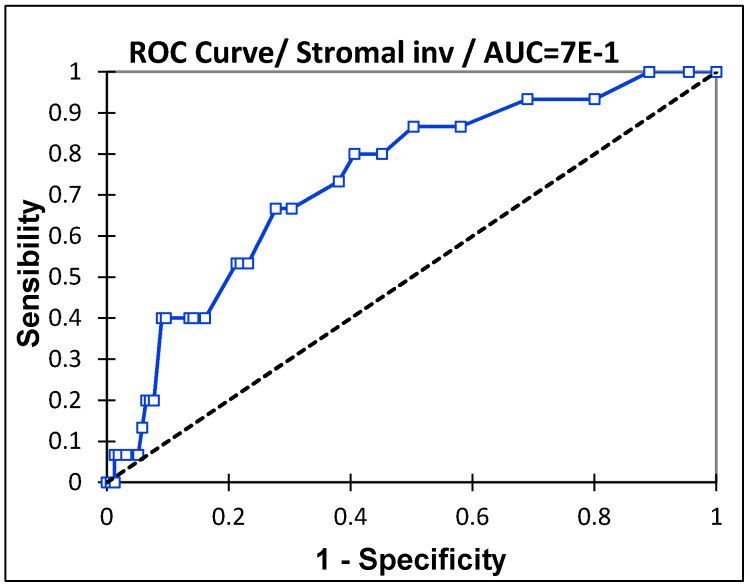
A Receiver Operating Characteristic (ROC) curve showing the association with stromal invasion and the presence of low-volume metastasis.

**Figure 2:**
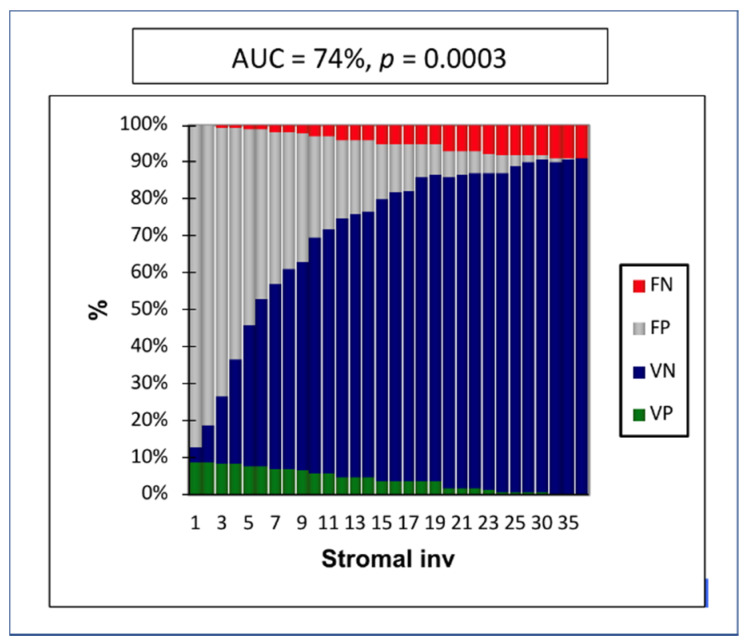
Stromal invasion and the presence of low-volume metastasis. FN = false negative, FP = false positive, VN = true negative, VP = true positive, AUC = Area under the Curve.

In the Receiver Operating Characteristic (ROC) curve, the depth of stromal invasion was the strongest prognostic factor, with an Area under the ROC curve (AUC) of 0.74 (IC 95%: 0.61–0.86, *p* = 0.0003) (Figure 1). The maximum specificity and sensitivity were found at a cutoff of 8 mm. The negative predictive value (NPV) in cases of stromal invasion < 8 mm was 97% (Figure 2).

This cutoff remains the best prognostic factor for all types of lymph node metastases (MAC, MIC, and ITC).

For this reason, we decided to analyze the effect of stromal invasion according to three groups. The first group included patients with ≤ 3 mm of invasion (low risk according to the new FIGO 2018 classification [1]), the second with invasion of 3.1–8 mm (intermediate risk), and the third group, with invasion > 8 mm (high risk). 

The univariate analysis of risk factors, tumor type, pathological stage, grade, size, stromal invasion, and lymphovascular space invasion (LVSI), showed a significant correlation between the presence of low-volume metastasis, stage > IB1 (*p* = 0.02) and stromal invasion > 8mm (*p* = 0.01). None of the histological tumor types in our sample (squamous cells, adenosquamous and adenocarcinoma) seemed to be associated with the presence of low-volume metastases. The results are shown in Table 2.

**Figure 3:**
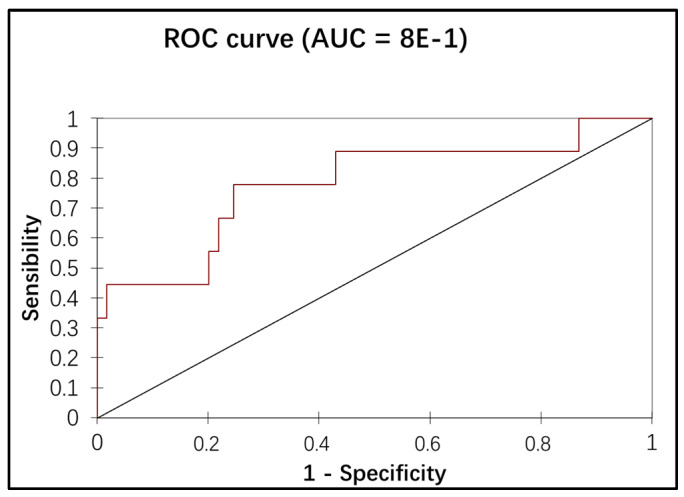
A ROC curve showing the association of stage and lymphovascular space invasion (LVSI) with the presence of low-volume metastasis using logistic regression. The discriminating power of low-volume metastasis detection: the association of two factors (Stage + LVSI) in the logistic regression. ROC = Receiver Operating Characteristic, AUC = Area under the Curve.

Variables with p values lower than 0.1 by univariate analysis were entered into a multivariate logistic regression model. Multivariate analysis showed a significant correspondence between the presence of low-volume metastasis, the IB1 stage, and positive LVSI (Table 3). The association of the stage and the LVSI in the multiple logistic regression showed an additional discriminating power of low-volume metastasis detection (the AUC of the ROC curve = 78%), as illustrated in Figure 3.

### 2.3. Predictive Factors of Negative Node

**Figure 4:**
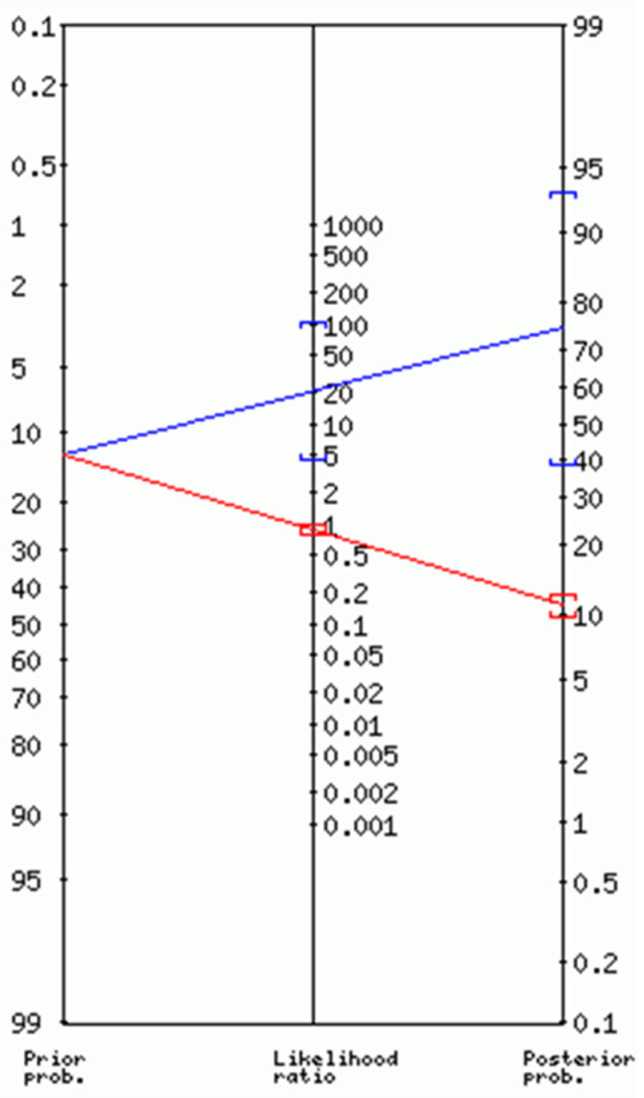
Predictive test of N0 patients in absence of parametrial and lymphovascular invasion. Positive test: blue; Negative test: red; Odds = Probability/ (1 − Probability); +LR = Sensitivity/(1 − Specificity); −LR = (1 − Sensitivity)/Specificity; Posterior Odds = Prior Odds × LR.

In our group, we observed a correlation between lymph node invasion (MAC, MIC, and ITC) and higher than IB1 stage (*p* = 0.03), tumor size > 2 cm (*p* = 0.05), stromal invasion > 8 mm (*p* = 0.05), and positive LVSI (*p* = 0.0005) in the univariate analysis. In the multivariate analysis, stage IIB (*p* = 0.02) and LVSI (*p* = 0.003) remained significant. If we consider these two risk factors of lymph node invasion in the multivariate analysis, we can create a predictive test of negative node (N0). In the absence of parametrial and lymphovascular invasion, the negative likelihood ratio was 0.86 (95% CI: 0.75–0.98) with a posterior probability (posterior odds) of 11% (95% CI: 10–12%), meaning that about 1 in 1.1 patients with a negative test was genuine N0 (Figure 4).

In N0 situation, the depth of stromal invasion with the highest sensitivity and specificity is 32 mm with a specificity of 100%, a positive predictive value (PPV) of 100%, and 0 false positive (FP) cases. All patients with stromal invasion > 32 mm also had positive LVSI.

### 2.4. Disease-Free Survival

**Figure 5:**
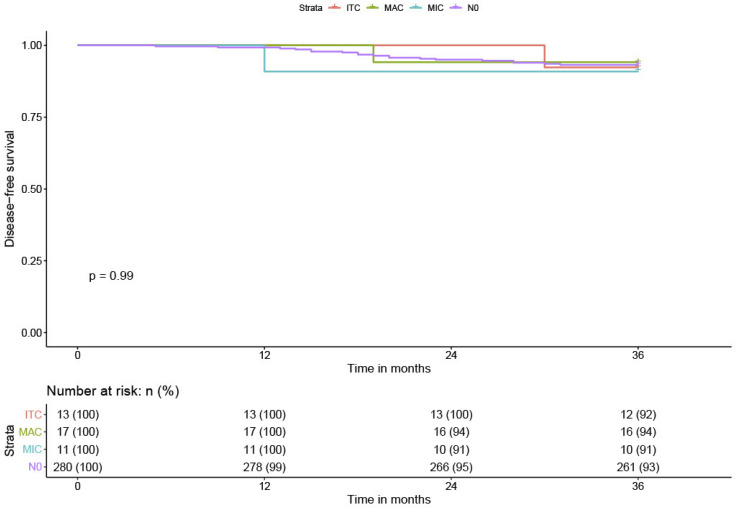
Three-year disease-free survival (DFS) and lymph node status.

Twenty-one patients (6.5%) experienced recurrence during the follow-up: nine vaginal recurrences, seven distant metastases (lung, liver, and bladder), and five with lymph node involvement. The overall DFS was 93.5% at three years (0.93; CI 95%: 0.90–0.96). No patients with positive lymph nodes had a lymphatic recurrence. Among the 24 patients with low-volume metastases, one patient with ITC had bladder metastasis and one patient with MIC had vaginal recurrence. No significant difference was found between patients with low-volume metastases (DFS = 92.7% (0.93; CI 95%: 0.90–0.96)) compared to N0 patients (DFS = 93.6% (0.93; CI 95%: 0.90–0.96)). The DFS in MIC patients was 91% (0.91; CI 95%: 0.88–0.94), the DFS in ITC patients was 92.4% (0.92; CI 95%: 0.89–0.95). The DFS in lymph node positive for MAC patients was similar, 93% (0.93; CI 95%: 0.91–0.95) (Table 1 and Figure 5). All patients with MAC were treated with radiochemotherapy.

The median time to recurrence in patients with low-volume metastases was 21 months (12–30 months); in patients with MAC, 19 months (only one case); and in the N0 patients, 19.5 months (5–31 months). While these differences were not considered statistically significant, the time to recurrence tended to be longer in the low-volume metastases group than in the node-negative group.

### 2.5. Low-Volume Metastasis Treatment

Concerning low-volume metastasis, 13 patients received adjuvant treatment and 11 patients did not. In the Senticol 1 study, only patients with other risk factors, according to Sedlis criteria [13], were treated with adjuvant therapy. Patients with low-volume metastasis who were included in the Senticol 2 study systematically received adjuvant treatment, except one patient with ITC, who declined additional treatment and did not experience recurrence. In fact, adjuvant treatment in the case of MIC and ITC was significantly more frequent in Senticol 2 compared to Senticol 1 (*p* = 0.003). The characteristics of patients with low-volume metastasis are shown in Table 3. There was no statistical difference in the DFS in low-volume metastasis patients treated with radiochemotherapy, in comparison to those without adjuvant treatment: among 11 patients who did not receive radiochemotherapy, one patient had recurrence (1 MIC case), and among the 13 patients treated by radiochemotherapy, one patient had recurrence (1 ITC case).

**Figure 6:**
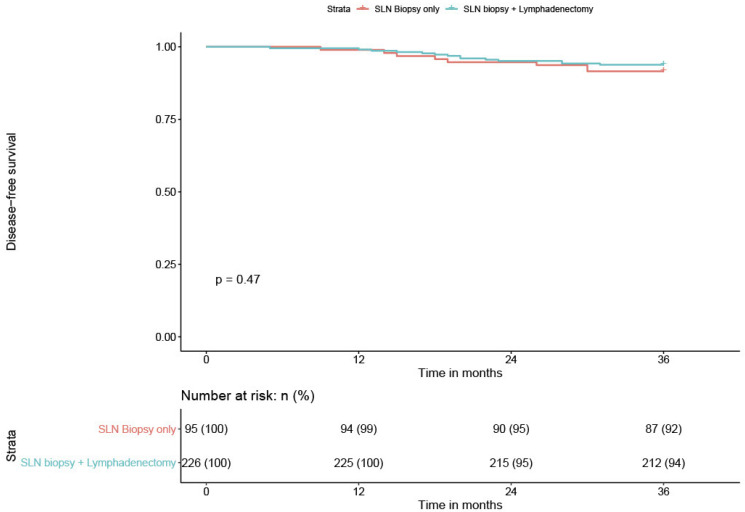
DFS of low-volume metastasis patients according to the type of nodal staging. SLN = sentinel lymph node.

We compared the DFS of low-volume metastasis patients according to the type of nodal staging, sentinel biopsy alone (SLN) versus sentinel biopsy plus lymphadenectomy (SLN + PLND). We recorded one recurrence among the six low-volume metastasis patients who had a sentinel biopsy alone for a patient with ITC. All six patients had an adjuvant treatment. Among the 17 low-volume metastasis patients who had SLN + PLND, we had only only recurrence. This patient presented with MIC and did not receive adjuvant treatment. The difference was not statistically significant (*p* = 0.45); however, there was a trend in favor of SLN + PLND. The recurrence rate in the case of low-volume metastasis was too low to draw robust conclusions (Table 4 and Figure 6).

### 2.6. The Risk Factors of Recurrence 

According to the Cox proportional risk model, none of the considered risk factors (age, histological type like squamous cells, adenocarcinoma or adenosquamous cells, stage, stromal invasion, size, degree, or LVSI) had a significant correlation with the presence of recurrence after three years.

It is interesting to consider that the number of lymph node metastases did not change the prognosis (no difference in DFS).

The bilateral or unilateral lymph node metastasis did not appear to influence the DFS—the two patients who had bilateral MAC developed no recurrence.

## 3. Discussion

In this study, we analyzed the risk factors associated with lymph node metastasis. We focused on cervical stromal invasion and demonstrated that the cutoff of 8 mm was the best prognostic factor for MAC, MIC, and ITC. For this reason, in this study, we considered patients in three groups, according to the stromal invasion. The first group included patients with ≤ 3 mm of invasion (low risk), the second one with invasion of 3.1–8 mm (intermediate risk), and the third one with invasion > 8 mm (high risk). We propose considering an 8 mm cutoff for the identification and classification of risk categories, rather than middle or deep third stromal invasion criteria, as suggested by Seidlis [13]. In our population, if the stromal invasion was > 32mm, we always found lymph node metastasis.

Furthermore, a diagnostic test that associates the absence of parametric and lymphovascular invasion appears to be a good prognostic test for N0.

The LACC study [14] is the trial that recently changed our surgical practice in early-stage cervical cancer. A comparaison with Ramirez’s study is therefore inevitable. The lymph-node involvement in LACC trial was 12.4% in laparoscopy (LPS) group and 13.1% in laparotomy (LPT) group. In our study, the lymph node involvement (MIC, MAC and ITC) is similar to LAAC (41 N+ / 321 patients = 12.7%). 

Low-volume metastasis treatment is a subject of debate. MIC and ITC are generally found in definitive histological analyses and rarely in intraoperative analyses as they usually require ultrastaging. To date, low-volume metastases, in particular MIC, have been considered as true metastases, prompting adjuvant treatment. Hence, in the case of low-volume metastasis, we currently include radiochemotherapy treatment [9], even if lymphadenectomy was undertaken before, with significantly increased morbidity. Our recent trial [5] questioned adjuvant treatment in the case of low-volume metastasis. Another prospective article [6] confirmed our results, disproving the hypothesis that low-volume metastasis had a deleterious impact on the recurrence risk. The presence of MIC and ITC did not appear to influence the elapsed time before recurrences; indeed, the time without disease appeared longer than in the case of N0 and MAC. We cannot explain this fact, which is also common with other diseases, for example, endometrial cancer [15].

This study confirmed the hypothesis supported in our previous article [5]: the presence of MIC and ITC did not reduce the DFS and the addition of an adjuvant treatment did not improve the disease-free survival after three years. The only three prospective studies [5,6] and this study, therefore, appear to suggest that adjuvant treatments should be de-escalated in cases of MIC and ITC in the absence of other risk factors. 

In the histopathological analysis, ITC and MIC were considered as different entities. Unlike the ITCs, MICs are a small collection of cancer cells that shed from the original tumor and spread to another part of the body through the lymphovascular system. Currently, these two entities are treated in the same way. As their true impact remains currently unknown, it is customary to use adjuvant treatment in cases of both MIC and ITC. However, the question can be raised as to the adjuvant treatment: is it the correct approach? Considering the results of the prospective studies, we propose that the addition of the adjuvant treatment is not appropriate. The decision should be made considering other risk factors and after a multidisciplinary discussion between oncologists, radiotherapists, gynecologists, and the patient, without forgetting that the future treatment will likely be immunotherapy.

In this analysis, we found a favorable trend in patients with low-volume metastasis treated by lymphadenectomy in comparison to patients with sentinel biopsy alone. This result seemed to confirm the study of Zaal [16] which lends support to the observation that survival is significantly improved (*p* = 0.046) by lymphadenectomy (>16 lymph nodes) when the SLN is positive for MIC or ITC. At the moment, we cannot confirm that hypothesis due to the low number of recurrences in cases of low-volume metastasis; however, it remains a suggestion for further study. Considering the high DFS in our patients, we would also require a very large number of patients with low metastases to be able to confirm the raised hypotheses.

In comparison with the LACC [14], we obtained a much higher DFS in general (93.5%), especially regarding the patients operated by LPS. In fact, if we consider only the radical hysterectomies and the stage IA1 LVSI+, IA2 and IB1 (like in the LAAC trial), among the 191 radical hysterectomies, patients who underwent surgery by LPT had a significant decrease in DFS (*p* = 0.05) in comparison with minimally invasive surgery, but there is likely a selection bias, because the surgical choise in our study (conceived before LAAC) was the LPS and we performed LPT only in case of technical or intra-operative difficulty.

A large randomized prospective study should be carried out to answer these questions. We look forward to obtaining further answers from the Senticol 3 trial [17].

## 4. Materials and Methods

### 4.1. Patient Selection

In this study, we included all Senticol 1 [10] and Senticol 2 [11] patients. The pre-operative staging was performed with clinical examination, diagnostic biopsy or conisation, pelvic MRI, total body PET-CT scan or thoraco-abdominal CT scan. An inclusion criterion of Senticol 1 and Senticol 2 studies was the FIGO 2009 [18] stage IA1 LVSI+, IA2 and IB1. The stage was defined by pre-operative assessment. In definitive histological analysis 19 patients were > IB1 in reason of tumor larger than 4 cm or presence of minimal parametrial invasion, not suspected until the final result.

We performed a hysterectomy or trachelectomy in 259 patients.

A type B radical hysterectomy (Querleu-Morrow) [19] was performed in 172 patients, a type C radical hysterectomy was performed in 23 patients, a simple hysterectomy was performed in 5 cases, a radical trachelectomy (fertility-sparing surgery) was performed in 55 patients, a simple trachelectomy in 4 cases. Of note, all patients underwent identification of the SLN by laparoscopy; however, 21 patients underwent a laparotomy, 77 had simple laparoscopic approach, 140 vaginal-assisted laparoscopy and the remaining 21 patients underwent vaginal trachelectomy.

Written informed consent was obtained from all patients. The follow-up data of included patients were censored after 3 years, like defined in the protocol of Senticol 1 and 2. Standard, guideline-based clinical follow-ups (every 3–4 months with clinical examination and eventual Pap test) were performed for 3 years. The DFS was evaluated with respect to the nodal status, histologic type, tumor diameter, age, presence of LVSI, and preoperative brachytherapy treatment.

Senticol 1 [10] is a prospective longitudinal study that was conducted in seven centers in France between January 2005 and June 2007. Patients were enrolled prospectively and followed for 3 years. In Senticol 1, all patients underwent lymphadenectomy after laparoscopic identification of the SLN. A revision of the histologic sections of the lymph nodes and ultrastaging was performed 3 months after the original surgery in two pathology reference centers on all lymph nodes (sentinel and non-sentinel). The reason for performing ultrastaging on all nodes was to be certain that the negative predictive value of the SLN technique was adequate and that MICs or ITCs in the non-sentinel nodes were not missed. The discovery of MICs or ITCs in the secondary ultrastaging analysis did not alter the adjuvant treatment as these data were obtained more than 3 months after the surgical treatment.

Senticol 2 [11] is a multicenter prospective randomized controlled trial comparing SLN biopsy alone to combined SLN biopsy and lymphadenectomy in early cervical cancer. Patients with a history of laparoscopic surgery and SLN biopsy in cervical cancer were recruited from 28 French centers between the period of March 2009 and June 2012. Randomization was performed during surgery (SLN only vs. SLN + PLND). Inclusion criteria were the bilateral detection of the SLN (isotopic + colorimetric), lymphoscintigraphy results, the bilateral intraoperative identification of SLN, and negative assessment based on SLN. Dynamic, balanced open-label randomization stratified by center with a 1:1 allocation using a block size of four was performed during the surgery.

In Senticol 1 [10], low-volume metastases were postoperatively treated with radiochemotherapy only in the presence of other concomitant risk factors; however, patients had systematic lymphadenectomy. On the contrary, in Senticol 2 [11], all positive lymph node patients (including MIC and ITC cases) had recommended adjuvant radiochemotherapy treatment.

Survival in the early stages of cervical cancer is very high, so in this study, we decided to focus mainly on DFS. We analyzed the impact of MIC and ITC on DFS, considering also other risk factors associated with low-volume metastasis and with cervical cancer. 

### 4.2. Sentinel Lymph Node Detection

The radioactive tracer colloidal rhenium sulfide labeled with technetium (99mTc) (Nanocis, 120MBq, Cis Bio International, Gif sur Yvette, France) and then 2.5% Patent Blue (2 mL diluted in 2 mL saline; Bleu Patenté V sodique, Guerbet, Roissy, France) were injected into the cervix at the 3, 6, 9, and 12 o’clock positions. The lymphoscintigram was required. 

Intraoperatively, the pelvic and paraaortic nodes were examined. SLNs were defined as blue-stained and/or radioactive lymph nodes or lymph nodes having a bluish afferent channel. SLNs were selectively extracted in a bag, their radioactivity being noted before and after extraction. The absence of residual radioactivity was confirmed in NSLN left in situ.

### 4.3. Pathological Lymph Node Analysis

All lymph nodes underwent histologic examination. SLNs were sectioned every 200 µm and were stained with hematoxylin-eosin-saffron (HES). When staining was negative, a section from the same level was examined by using IHC with the pancytokeratin antibody AE1-AE3 (DAKO, Trappes, France). NSLN were sectioned once and were examined by HES. Ultrastaging analysis was performed for all NSLN in 141 patients (44% of patients).

### 4.4. Data Collection

All data were prospectively entered into a database. 

### 4.5. Statistical Methods

Standard summary statistics were used, and a chi-squared test was applied to assess the categorical data. A value of *p* = 0.05 was used as the limit of statistical significance in all other parametric analyses. The Kaplan–Meier method was used to describe DFS. DFS was calculated as the time interval between the time of surgery and the time when disease recurrence was identified. A log-rank test was applied to compare the survival in different groups of patients in stratified survival analyses.

The association with low-volume metastasis and the other risk factors was analyzed with the chi-square test with a univariate model. Variables with *p* values lower than 0.1 by univariate analysis were entered into a multivariate logistic regression model in order to determine variables that were independently associated with low-volume metastasis. The discriminating power of risk factor associated with low-volume metastasis was calculated with a ROC curve.

The correlation between recurrences and risk factors was calculated using the Cox proportional hazards model.

The data were recorded in an Excel file and all statistical analyses were performed using XLStat Biomed software (AddInsoft 2020). Figure 5 and Figure 6 were made with RStudio program, Version 1.2.5042 2009–2020.

## 5. Conclusions

The results of this study confirm our previous analysis of Senticol 1 patients [5]: the presence of low-volume metastasis did not increase the DFS in early-stage cervical cancer patients (IA1 LVSI+, IA2 and IB1 according to FIGO 2009). We consider that the addition of the adjuvant treatment to the surgery in case of low-volume metastases is not appropriate. The decision of treatment in case of low-volume metastasis should be made considering other risk factors and after a multidisciplinary discussion between oncologists, radiotherapists, gynecologists, and the patient, without forgetting that the future treatment will likely be immunotherapy.

## Figures and Tables

**Table 1:** Patients definitive lymph nodal status.

LN Status	Observed	Recurrence	%	DFS
ITC	13	1/13	7.7%	92.4%
MIC	11	1/11	9%	91%
MAC	17	1/17	5.9%	93.8%
N0	280	18/280	6.4%	93.6%
Total	321	21/321	6.5%	93.5%

LN status: lymph nodal status; ITC: isolated tumor cells; MIC: micrometastasis; MAC: macrometastasis; N0: negative node; DFS: disease free survival.

**Table 2:** The factors associated with the presence of low-volume metastasis versus N0.

Variables	Total Number	Frequency with MIC/ITC %	Frequency with N0%	*p*(Chi-Square) Univariate
Histological type				
Squamous	208	7.2%	92.8%	
Adenocarcinoma	85	8.2%	91.8%	
Adenosquamous	7	14%		0.77
Stage				
IA1 LVSI +	13	0	100%	
IA2	16	6%	94%	
IB1	253	7%	93%	
>IB1	19	26%	74%	0.02
Grade				
G1	95	5%	95%	
G2	77	6.5%	93.5%	
G3	35	2.5%	8.5%	0.61
Size				
<2 cm	185	5.4%	94.6%	
>2 cm	96	11.4%	88.6%	0.07
Stromal invasion				
≤3 mm	32	3%	97%	
4–8 mm	82	4.8%	95.2%	
>8 mm	58	17%	73%	0.01
LVSI				
positive	77	7%	93%	
negative	223	13%	87%	0.06

MIC = micrometastasis, ITC = isolated tumor cells, N0 = negative lymph node, LVSI = lymphovascular space invasion.

**Table 3:** Multivariate analysis of the factors associated with the presence of low-volume metastasis.

Variables	OR	IC (95%)	*p* (Chi-Square) Multivariate
Stage			
IA1 L+	0.14	0.003–5.68	0.30
IA2	0.70	0.02–27.98	0.84
IB1	0.16	0.03–0.78	0.02
>IB1	1		
LVSI			
LVSI+	6.23	1.36–28.45	0.02
LVSI−	1		

LVSI = lymphovascular space invasion.

**Table 4:** Low-volume metastasis DFS considering lymph nodal surgery

Type	Number	Recurrence	Safe
**SLN**	6	1	5
**SLN + PLND**	17	1	16

SLN = sentinel biopsy alone; SLN + PLND= sentinel biopsy + lymphadenectomy.
